# Effect of precipitation change on the photosynthetic performance of *Phragmites australis* under elevated temperature conditions

**DOI:** 10.7717/peerj.13087

**Published:** 2022-03-10

**Authors:** Linhong Teng, Hanyu Liu, Xiaonan Chu, Xiliang Song, Lianhui Shi

**Affiliations:** 1 Dezhou University, Dezhou, China; 2 Shandong Agricultural University, Taian, China

**Keywords:** *Phragmites australis*, Photosynthesis, Precipitation, Warming, Non-stomatal limitation, Protection mechanism

## Abstract

**Background:**

As a fundamental metabolism, leaf photosynthesis not only provides necessary energy for plant survival and growth but also plays an important role in global carbon fixation. However, photosynthesis is highly susceptible to environmental stresses and can be significantly influenced by future climate change.

**Methods:**

In this study, we examined the photosynthetic responses of *Phragmites australis* (*P*. *australis*) to three precipitation treatments (control, decreased 30%, and increased 30%) under two thermal regimes (ambient temperature and +4 °C) in environment-controlled chambers.

**Results:**

Our results showed that the net CO_2_ assimilation rate (*P*_n_), maximal rate of Rubisco (*V*_cmax_), maximal rate of ribulose-bisphosphate (RuBP) regeneration (*J*_max_) and chlorophyll (Chl) content were enhanced under increased precipitation condition, but were declined drastically under the condition of water deficit. The increased precipitation had no significant effect on malondialdehyde (MDA) content (*p* > 0.05), but water deficit drastically enhanced the MDA content by 10.1%. Meanwhile, a high temperature inhibited the positive effects of increased precipitation, aggravated the adverse effects of drought. The combination of high temperature and water deficit had more detrimental effect on *P*. *australis* than a single factor. Moreover, non-stomatal limitation caused by precipitation change played a major role in determining carbon assimilation rate. Under ambient temperature, Chl content had close relationship with *P*_n_ (R^2^ = 0.86, *p* < 0.01). Under high temperature, *P*_n_ was ralated to MDA content (R^2^ = 0.81, *p* < 0.01). High temperature disrupted the balance between *V*_cmax_ and *J*_max_ (the ratio of *J*_max_ to *V*_cmax_ decreased from 1.88 to 1.12) which resulted in a negative effect on the photosynthesis of *P*. *australis*. Furthermore, by the analysis of Chl fluorescence, we found that the xanthophyll cycle-mediated thermal dissipation played a major role in PSII photoprotection, resulting in no significant change on actual PSII quantum yield (*Φ*_PSII_) under both changing precipitation and high temperature conditions.

**Conclusions:**

Our results highlight the significant role of precipitation change in regulating the photosynthetic performance of *P*. *australis* under elevated temperature conditions, which may exacerbate the drought-induced primary productivity reduction of *P*. *australis* under future climate scenarios.

## Introduction

Global warming mainly caused by high levels of greenhouse gas emission is predicted to increase the air temperature by 1.1–6.4 °C in the next hundred years (Crowther et al., 2016). At the same time, extreme precipitation events like drought and waterlogging will occur more universally than ever (IPCC, 2019). The changing global climate will not only aggravate the frequency and intensity of environmental stresses but also pose serious threat n agriculture production (Hossain et al., 2021; Vaughan et al., 2018; Xin & Tao, 2021), ecosystem stability (Kanojia & Dijkwel, 2018; White et al., 2021) and terrestrial C and N cycling (Crowther et al., 2016; Li et al., 2021b). Among the environmental factors, ambient temperature and soil water content are two major abiotic factors in the limitation of plant distribution and productivity (Küsters et al., 2021; Yan, Zhong & Shangguan, 2020; Kumari et al., 2021). Their change will directly and/or indirectly influence plant physiological processes, such as resource allocation (Farfan-Vignolo & Asard, 2012; Forbesa et al., 2020), net photosynthetic rate (Shao et al., 2021; Yamori, Hikosaka & Way, 2014), carboxylation efficiency (Liu et al., 2022), photochemical efficiency of photosystem II (PSII) (Aragón-Gastélum et al., 2020; Song et al., 2016a) and water use efficiency (Liu et al., 2019), which then impact the global carbon cycling. Among all the plant physiological processes, photosynthesis plays an important role in substance metabolism (Ort et al., 2015; Zhu et al., 2020). Thus, the understand of how plant photosynthesis responses to the concurrent warming and precipitation change is necessary for plants better facing future climate change.

The high limitation on the plant carbon assimilation capacity under soil water deficient conditions has been a major reason for plant growth and crop productivity reduction (Hussain et al., 2021; Nolf et al., 2015). It is widely accepted that there are two ways in which water stress affects the photosynthesis of plants: one is the stomatal limitations, such as closing the stoma and lowering the stomatal conductance (Daryanto, Wang & Jacinthe, 2017; Talbi et al., 2020); the other is non-stomatal limitations, such as photosynthetic phosphorylation (Du et al., 2021), regeneration of ribulose-1,5-bisphosphate (RuBP) (Song et al., 2016a), activation of Rubisco and the synthesis of ATP (Ashraf & Harris, 2013; Hu et al., 2020). The stomatal limitation is generally considered as the main factor responsible for the reduction of photosynthesis under drought stress environment (Liu et al., 2005; Song et al., 2020). However, long term of drought stress may lead to the reduction of chlorophyll content (Bijanzadeh, Barati & Egan, 2022), the content of Rubisco (Gadzinowska et al., 2021), the maximum Rubisco carboxylation rate and potential maximum rate of electron transport for RuBP regeneration (Song et al., 2016a), resulting in the decline of the plants’ photosynthetic rate (He et al., 2021; Wang et al., 2019).

The photosynthesis of plants is regarded as the most sensitive process to high temperature stress (Xalxo et al., 2020). High temperature lasting for only a few minutes to several hours will drastically damage the structure and function of photosynthetic apparatus such as thylakoid lamella and stroma, decrease the production of ATP, inhibiting a series of enzyme activities, affect the transport of photosynthetic electrons and reduce the photosynthetic rate finally (Hu et al., 2020). Heat stress can also cause photosynthesis decline through enhancing the generation of reactive oxygen species (ROS) (Hao et al., 2019), destroying the function of PSII (Jahan et al., 2021; Janka et al., 2015) suppressing the synthesis of chloroplast (Song, Wang & Lv, 2016), and inhibiting the activity of ribulose1,5-bisphosphate carboxylase/oxygenase (Rubisco) (Perdomo et al., 2017). In tomato plants, heat stress (40 °C) significantly decreased photosynthetic pigment concentrations and inhibited Rubisco accumulation resulting in a reduction of photosynthetic efficiency (Parrotta et al., 2020). Based on a 3-year study, Zhong et al. (2014) also reported that an air temperature elevation of 1.5 °C could decreased the net photosynthetic rate of *Phragmites australis* by 28%. In contrast, a recent study showed that increase of 4 °C significantly increased the net photosynthesis rate, transpiration rate, leaf temperature and chlorophyll content in leaves of lettuce by 114.9%, 65.5%, 7.1% and 9.8%, respectively (Ouyang et al., 2020). Although an emerging pool of knowledge shows that plant photosynthesis was noticeable affected by heat stress, the mechanism of the photoinhibition caused by high temperature is still need further research.

Coastal wetlands account for 0.22–0.34% of global land surface (Fennessy, 2014) and act as “blue carbon” resources due to the relatively high net primary productivity and low organic matter decomposition rate (Drake et al., 2015; Zhong et al., 2016). It is estimated that 13–17.2 Pg of carbon were stored in coastal wetlands (Hiraishi et al., 2014). However, coastal wetlands are also potential source of global greenhouse gases (Hsieh et al., 2020). The climate change increased the release rate of carbon in the CO_2_ and CH_4_ through organic matter decomposition and decreased the amount of carbon stored in coastal wetlands. It is found that a 1.5 °C temperature enhancement could result in the gas emissions released form wetlands increase by 37.5% (Liu et al., 2020). As plant photosynthesis is the major way of carbon fixation in coastal wetlands, keep the photosynthesis at a high rate under climate change conditions is essential for global carbon cycling. *Phragmites australis* (*P. australis*) belonging to the Poaceae family, is the main constructive and dominant plants in coastal wetlands of China and plays an important role in maintaining the ecosystem function (Guan et al., 2017). Their spatial distribution is mainly limited by air temperature change and soil water deficit. The research on *P. australis*’s photosynthetic characteristics in response to rising temperature and changing precipitation pattern can provide a theoretical basis for dealing with climate change in coastal wetlands. This main aims of the work were to investigate the photosynthetic responses of *P. australis* to precipitation change under elevated temperature conditions. Specifically, three key research questions were addressed in the paper: (1) Are there any negative or positive influences of temperature and precipitation change on photosynthetic performance of *P. australis*? (2) What are the physiological mechanisms of precipitation change and high temperature affecting the carbon assimilation of *P. australis*? (3) What are the protection mechanisms of *P. australis* to avoid damage caused by environmental stress?

## Materials and Methods

### Plant culture and experimental design

The experiment was carried out at the Dezhou University, Shandong Province, China. The seeds of *P. australis* and soils were obtained from the costal wetland in Kenli, Dongying, China. The soil sample site has a northern subtropical marine monsoon climate. The annual average temperature and precipitation which obtained from the Kenli Meteorological Station of the China Meteorological Administration (37°35′N, 118°33′E; elevation 85 m) in the past 10 years (2010–2019) were 12 °C and 552 mm, respectively. About 70–74% of the annual precipitation is concentrated from July to September.

Before sowing in plastic pots, the seeds of *P. australis* were sterilized by potassium permanganate solution (0.7%) for 8 min and washed with deionized water for three times. Each plastic pot (18 cm in height and 20 cm in diameter) was filled with 5.0 kg of dry soil and planted with 10 plants. The experimental soil was paddy fluvo-aquic soil, and the basic physical and chemical properties of the soil were as follows: soil pH 7.91, organic matter 9.42 g·kg^−1^, total nitrogen 0.77 g·kg^−1^, available phosphorus 5.92 g·kg^−1^, and available potassium 168.72 g·kg^−1^.

After the third leaf emerged, the seedlings were thinned to three plants per pot. There were three precipitation treatments and two temperature treatments were selected for experiment. The precipitation treatments were set as: average monthly precipitation (July to September) over 10 years (W_0_); W_0_ increased by 30% (W_+30_); W_0_ decreased by 30% (W_−30_). The temperature treatments were set as 26.3/21.6 °C (T_0_) and 30.3/25.6 °C (T_4_). The treatments were set based on the monthly average temperature and rainfall during *P. australis*’s major growth stage (July to September) in the past 10 years (2010–2019). Each treatment and corresponding experiments were established in triplicates. Totally, 18 pots with healthy plants (three plants per pot) were randomly selected and placed into two environmental control chambers (RGD-500D3). The size of environmental control chamber was 750 × 660 × 2,050 mm (length × width × height). Growing conditions in the environmental control chamber were maintained as follows: 390 ppm CO_2_ concentration, 1,000 µmol photons·m^−2^·s^−1^ photosynthetic photon flux density, and 14 h photoperiod per day. All the parameter measurements were conducted after 92 days of plant growth.

### Measurements

#### Leaf gas exchanges

Three plants from each treatment were randomly chosen from different pots for measurement. Gas exchange parameters were measured on the healthy and fully expanded leaves of *P. australis* with an open gas exchange system (CIRAS-3, PP-system, Hitchin, UK). Illumination was supplied to the leaves from a red-blue LED light source. The leaf chamber temperature, CO_2_ concentration and photosynthetic photon flux density (PPFD) were controlled at 25 °C, 390 ppm and 900 μmol·m^−2^·s^−1^, respectively.

#### *A*/*C*_i_ curve

The measurement of *A*/*C_i_* curves was performed on the same leaves used for gas exchange parameter measurements. *A*/*C*_i_ curve was measured under a light saturation level of 900 μmol·m^−2^·s^−1^ PPFD, and estimated using the CO_2_ response curve of photosynthesis. The CO_2_ gradients for *A*/*C_i_* curves included 390, 200, 100, 50, 390, 600, 800, 1,000 μmol·mol^−1^ levels stepwise. The analysis of *A*/*C_i_* curve was conducted with using the plant ecophys R package, which based on the model of Farquhar, Von & Berry (1980).

#### Chlorophyll fluorescence measurements

Three areas of interest at different position of leaf were selected to calculate the fluorescence parameters. Based on the method described by Song et al. (2016b), the actual PSII quantum yield (*Φ*_PSII_), quantum yield of regulated energy dissipation of PSII (*Φ*_NPQ_), and quantum yield of nonregulated energy dissipation of PSII (*Φ*_NO_) were measured using an imaging-PAM fluorometer (Walz, Effeltrich, Germany). The fluorescence parameters were calculated using fellow equations described by Lazár (2015):

$$\eqalign{
 & {{\rm{\Phi }}_{{\rm{PSII}}}} = ({F_{\rm{m}}}\prime - {F_{\rm{s}}})/{F_{\rm{m}}}\prime = {\rm{\Delta }}F/{F_{\rm{m}}}\prime \cr 
 & {{\rm{\Phi }}_{{\rm{NPQ}}}} = 1 - {{\rm{\Phi }}_{{\rm{PSII}}}} - 1/[NPQ + 1 + q{\rm{L}}({F_{\rm{m}}}/{F_0} - 1)] \cr 
 & {{\rm{\Phi }}_{{\rm{NO}}}} = 1/[(NPQ + 1 + q({\rm{L}}))({{\rm{F}}_{\rm{m}}}/{{\rm{F}}_0}-1)] \cr} $$

where *F*_m_ is the maximum fluorescence in the dark-adapted state, *F*_0_ is the minimum Chl fluorescence yield, *F*_m′_ is the maximum fluorescence yield in the light-adapted state, *F*_s_ is the Chl fluorescence during actinic illumination, *q*L is the fraction of open PSII centers, *NPQ* is the non-photochemical quenching.

#### Chlorophyll content

The chlorophyll content was measured according to the method described by Hiscox & Israelstam (1979). Briefly, 0.25 g fresh leaf samples were mashed in 80% acetone (v/v) in a 4 °C refrigerator overnight. After filtered through two-layer nylon net, the extract was then centrifuged at 15,000 g for 5 min to obtain the supernatant. After determining the absorbance of the supernatant at wavelengths of 663 and 646 nm, the contents of chlorophyll a and b were calculated according to the equations of Lichtenthaler & Buschmann (2001):

$$\matrix{
 {{\rm{Chlorophyll a}} = 12.25{\rm{}}{{\rm{A}}_{663}} - 2.79{\rm{}}{{\rm{A}}_{647}}} \cr 
 {{\rm{Chlorophyll b}} = 21.50{\rm{}}{{\rm{A}}_{647}} - 5.10{\rm{}}{{\rm{A}}_{663}}} \cr 

 } $$

#### Malondialdehyde (MDA) content

The MDA content was measured according to the thiobarbituric acid (TBA) chromogenic method described by Song, Wang & Lv (2016). Briefly, 1.0 g fresh leaf samples were homogenized with 0.1% trichloroacetic acid (TCA, 2.0 mL, pH 7.0) for 2 h an 15,000 g for 10 min. Then, 0.5 mL of supernatant was added to 1.5 mL of TBA. After the mixture was incubated in a shaking water bath at 90 °C for 20 min, the reaction was rapidly stopped by ice-water bath. These samples were centrifuged at 10,000 g for 5 min to obtain the supernatant. The absorbance of the supernatant was detected at 532, 450, and 600 nm. The amount of MDA was calculated with the following equation:

$$MDA = 6.45 \times (A532 - A600) - 0.56 \times A450$$

### Statistical analysis

All statistical analyses were performed using SPSS 21.0 (SPSS Institute, Inc., Cary, NC, USA). Effects of warming and precipitation change were analyzed using one-way analysis of variance with a Duncan’s multiple range test at a 5% probability level. The linear curve fitting and graphing were performed using Origin 2021 software (Origin Lab, Northampton, MA, USA).

## Results

### Chlorophyll content

Under both two temperature conditions (T_0_ and T_4_), the Chl content of *P. australis* was significantly affected by precipitation change. It can be seen from Table 1 that, at the condition of T_0_, W_+30_ caused the increase of Chl a, Chl b and Chl a+b content by 25.6%, 33.8% and 31.1%, respectively, with the Chl a/b ratio decreased by 6.6%. At the same temperature, a decreasing precipitation (W_−30_) led to the decline in Chl b and Chl a+b content (10.1% and 6.2%, respectively) and the increase in Chl a/b ratio (12.2%). At a higher temperature (T_4_), the adjustment of precipitation resulted in similar variations in the contents and ratios of Chl contents. Moreover, under different precipitation conditions (W_+30_, W_0_ and W_−30_), the Chl a, Chl b and Chl a+b content at the higher temperature (T_4_) decreased by 3.5–13.0%, 18.7–32.0% and 12.9–24.2%, respectively, with the Chl a/b ratio increasing by 7.6–25.3%.

**Table 1:** Effects of warming and precipitation changes on chlorophyll content in leaves of *Phragmites australis*.

Treatment		Chl a (mg/g)	Chl b (mg/g)	Chl a/b (%)	Chl a + Chl b (mg/g)
T_0_	W_+30_	1.08 ± 0.05 a	1.86 ± 0.08 a	58.2 ± 0.7 b	2.95 ± 0.13 a
	W_0_	0.86 ± 0.04 b	1.39 ± 0.02 b	62.3 ± 1.7 b	2.25 ± 0.06 b
	W_−30_	0.86 ± 0.01 b	1.25 ± 0.09 c	69.9 ± 4.7 a	2.11 ± 0.10 b
T_4_	W_+30_	0.94 ± 0.01 a	1.51 ± 0.05 a	62.6 ± 2.4 c	2.45 ± 0.04 a
	W_0_	0.83 ± 0.02 b	1.13 ± 0.10 b	74.0 ± 4.4 b	1.96 ± 0.12 b
	W_−30_	0.75 ± 0.01 c	0.85 ± 0.04 c	87.6 ± 3.8 a	1.60 ± 0.05 c

**Note:**

Different lowercases indicate significant difference between different precipitation treatments within the same temperature treatment compared with control (*p* < 0.05).

### MDA content

**Figure 1:**
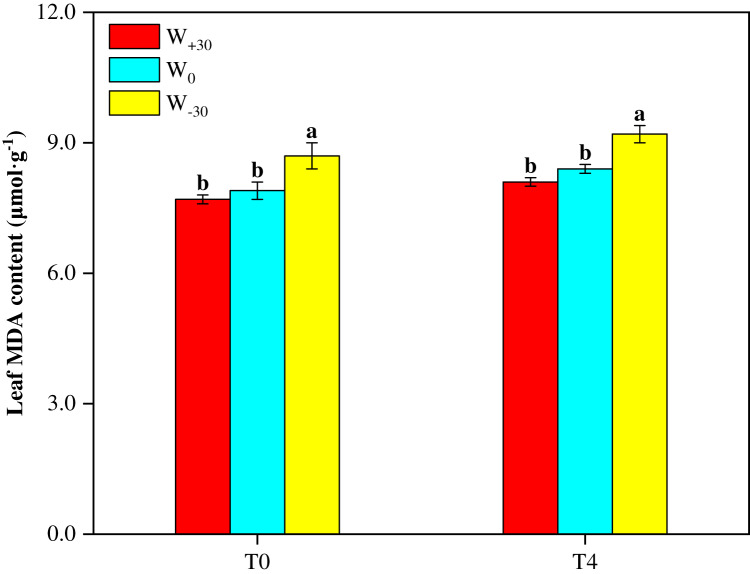
Effects of warming and precipitation changes on Malondialdehyde content in leaves of *Phragmites australis*. Vertical bars represent ±SD of the mean (*n* = 3), and different letters on the SD bars indicate significant differences among the all treatments (*p* < 0.05).

Malondialdehyde (MDA) as a product of lipid peroxidation can be used as a marker for oxidative stress under environmental stress conditions. The higher MDA content indicates the stronger cell membrane lipid peroxidation. It can be seen from Fig. 1 that, under both T_0_ and T_4_ conditions, W_+30_ had no significant effect on MDA content (*p* > 0.05). But W_−30_ led to the significant increase of MDA by 10.1% under T_0_ condition and by 9.5% under T_4_ condition. At the same time, high temperature also enhanced MDA content. As shown in Fig. 1, under different precipitation conditions (W_+30_, W_0_ and W_−30_), the MDA content in the T_4_ treatment groups increased by 5.2%, 6.3% and 5.7%, respectively, compared with the T_0_ treatment groups.

### Photosynthetic parameters

From Table 2, it was found that at the condition of T_0_, compared to W_0_, net CO_2_ assimilation rate (*P*_n_) in W_+30_ treatment increased by 32.8% and in W_−30_ treatment reduced by 18.9%, respectively. The other gas exchange parameters such as stomatal conductance (*G*_s_), intercellular CO_2_ concentration (*C*_i_), transpiration rate (*T*_r_) and water use efficiency (*WUE*) were not significantly affected by W_+30_ or W_−30_. At the condition of T_4_, the photosynthetic parameters between W_+30_ and W_0_ showed no remarkable difference, while W_−30_ significantly reduced the values of *WUE*, *P*_n_, *G*_s_, *C*_i_ and *T*_r_ by 25.2%, 52%, 14.1% and 33.0%, respectively. Under all precipitation conditions, high temperature negatively affected photosynthesis of *P. australis* and reduced *P*_n_ by 6.6∼17.4%.

**Table 2:** Effects precipitation change on photosynthetic parameters in leaves of *Phragmites australis* under ambient temperature (T_0_) and high temperature (T_4_) conditions.

Treatment		Photosynthetic parameters						
		*P*_n_ (μmol CO_2_·m^−2^·s^−1^)	*G*_s_ (μmol·mol^−1^)	*C*_i_ (mol H_2_O·m^2^·s^−1^)	*T*_r_ (mmol·m^−1^·s^−1^)	WUE (μmol CO_2_·mmol H_2_O)	*V*_cmax_ (μmol·m^2^·s^−1^)	*J*_max_ (μmol·m^2^·s^−1^)
T_0_	W_+30_	11.5 ± 1.0 a	0.18 ± 0.03 a	271 ± 12 a	3.5 ± 0.3 a	3.3 ± 0.3 a	52.0 ± 8.1 a	121.0 ± 23.1 a
	W_0_	8.6 ± 0.4 b	0.14 ± 0.08 a	224 ± 69 a	3.7 ± 1.8 a	2.7 ± 1.3 a	41.5 ± 1.8 b	75.5 ± 11.7 ab
	W_−30_	7.0 ± 0.7 c	0.15 ± 0.01 a	303 ± 2 a	3.4 ± 0.3 a	2.1 ± 0.4 a	27.1 ± 5.4 c	69.3 ± 18.6 b
T_4_	W_+30_	9.5 ± 0.1 a	0.14 ± 0.01 a	267 ± 2 a	3.5 ± 0.1 a	2.7 ± 0.1 a	58.1 ± 6.5a	110.6 ± 6.0 a
	W_0_	8.1 ± 0.7 a	0.17 ± 0.03 a	298 ± 15 ab	3.0 ± 0.3 a	2.7 ± 0.01 a	35.4 ± 4.6 b	79.0 ± 4.1 b
	W_−30_	6.0 ± 1.2 b	0.08 ± 0.01 b	256 ± 24 b	2.0 ± 0.6 b	3.2 ± 1.3 a	25.7 ± 3.8 b	56.5 ± 10.0 b

**Note:**

Different lowercases indicate significant difference between different precipitation treatments within the same temperature treatment compared with control (*p* < 0.05).

The change of *P*_n_ as a function of increased *C*_i_ in the chloroplast can be used to reflect the biochemical limitations of photosynthesis under high temperature and changing precipitation conditions. As shown in Table 2, at the condition of T_0_, W_+30_ enhanced *V*_cmax_ and *J*_max_ by 25.3% and 60.3%, while W_−30_ caused the reduction by 34.6% and 8.2%, respectively. At the condition of T_4_, W_+30_ resulted in a significant increase of *V*_cmax_ and *J*_max_ by 63.8% and 27.3%, while W_−30_ caused the reduction by 27.4% and 28.4%, respectively. Under W_0_ and W_−30_ conditions, T_4_ significantly reduced *V*_cmax_ by 14.5% and 5.1%, while increased *J*_max_ by 4.7% and 18.4%, respectively. At the condition of W_+30_, *V*_cmax_ increased by 11.7% and *J*_max_ decreased by 16.9% in the T_4_ treatment group.

### Chlorophyll fluorescence parameter

**Figure 2:**
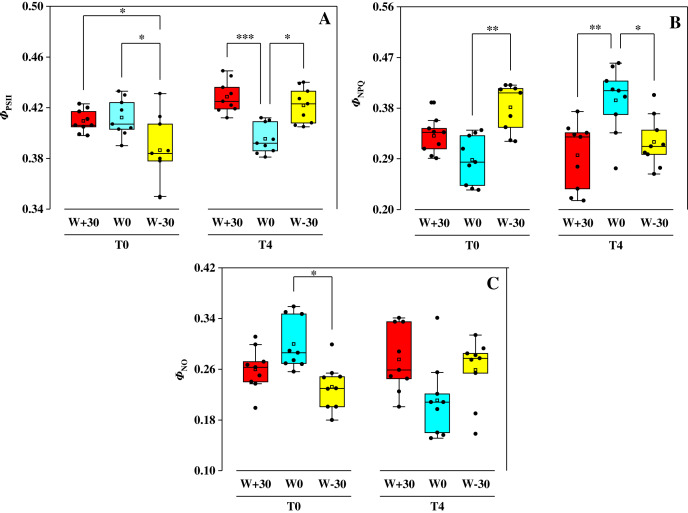
Effects of warming and precipitation changes on *Φ*_PSII_ (A), *Φ*_NPQ_ (B) and *Φ*_NO_ (C) in leaves of *Phragmites australis*. The horizontal line represents the median value and the open rectangle represents the mean value (*n* = 9). *p<=0.05, **p<=0.01, ***p<=0.001.

The effect of water treatments on *Φ*_PSII_, *Φ*_NPQ_ and *Φ*_NO_ under two temperature conditions were shown in Fig. 2. Under T_0_ condition, precipitation change (W_+30_, W_−30_) had no significant effect on *Φ*_PSII_ (*p* > 0.05), but drastically increased *Φ*_NPQ_ by 14.9% and 32.3% and reduced *Φ*_NO_ by 13.3% and 22.7%, respectively. Under T_4_ condition, *Φ*_PSII_ in the W_+30_ and W_−30_ treatment groups increased by 8.6% and 6.8%, *Φ*_NO_ increased by 30.3% and 21.3%, while *Φ*_NPQ_ decreased by 25.4% and 18.9%, respectively. Under different precipitation treatments (W_+30_, W_0_ and W_−30_), compared to T_0_, the change of *Φ*_PSII_ caused by T_4_ was 29.6%, −4.1% and 9.3%, the change of *Φ*_NPQ_ caused by T_4_ was −10.6%, 36.8% and −16.0%, and the change of *Φ*_NO_ caused by T_4_ was 5.8%, −29.7% and 11.6%, respectively.

## Discussion

High temperature and precipitation change as two major abiotic stresses always occur simultaneously, which threaten the sustainability of future crop production and biodiversity (Alam et al., 2021; Hosseini Sanehkoori, Pirdashti & Bakhshandeh, 2021; Küsters et al., 2021; Zhang et al., 2018). In the present study, we found that the positive effects of increased precipitation and the adverse effects of decreased precipitation on chlorophyll content, CO_2_ assimilation rate, lipid peroxidation (as indicated by MDA) and the energy partitioning of PSII were significant. Meanwhile, high temperature inhibited the positive effects of increased precipitation and aggravated the adverse effects of decreased precipitation. Similarly, in the studies on *Leymus chinensis* (Xu & Zhou, 2011), *Stipa bungeana* (Song et al., 2016b), *Ziziphus jujube* (Jiang et al., 2020), and *Robinia pseudoacacia* (Yan, Zhong & Shangguan, 2020), the high temperature combined with severe drought exacerbated the adverse effects on plant growth and photosynthesis.

Plants exposed to environmental stresses, such as drought, extreme temperatures or their combinations, have to face several metabolic imbalances leading to oxidative damage due to ROS accumulation, resulting in detrimental secondary effects on plant organelles (Raja et al., 2020; Vurukonda et al., 2016). ROS buildup in plants can damage cell functions by causing oxidative damage, resulting in DNA nicking, amino acids and photosynthetic pigments biosynthesis inhibition, and even cell death (Nath et al., 2016; Raja et al., 2017). MDA content, a result of ROS mediated lipid peroxidation, is used as biomarker of membrane damage caused by various abiotic stresses (Morales & Munné-Bosch, 2019). In the present study, increased precipitation showed no significant effect on MDA content in leaves of *P. australis*, while the decreased precipitation and elevated temperature remarkable increased the MDA content. The results suggest precipitation decreased by 30% and temperature elevated by 4 °C accelerates MDA formation, resulting in serious lipid peroxidation (Morales & Munné-Bosch, 2019). Similar results were found in studies on *Solanum lycopersicum* (Raja et al., 2020), maize (Naz et al., 2021), and *Echinacea purpurea* (Hosseinpour et al., 2020). The increase in the MDA content indicates that water deficit and high temperature destroy the antioxidant defense system, generate lipid peroxidation, and cause oxidative burst and excess oxidative damage to the cell membrane in *P. australis* plants. The increase in lipid peroxidation is widely reported to cause oxidative damage to chloroplast organs (Sohag et al., 2020) and leads to chlorophyll degradation (Bagheri, Gholami & Baninasab, 2019). The noticeable reduction of Chl a and Chl b in the W_−30_ and T_4_ treatment supports the finding that water deficit and high temperature trigger oxidative damage to the expression of chlorophyll a-b binding protein gene (Sun et al., 2022) and the synthesis of chlorophylls (Gujjar et al., 2020), which inevitably leads to a decrease in leaf photosynthetic efficiency (Wang et al., 2019) and plant productivity (Song, Jin & He, 2019).

The response of photosynthetic capacity to the variation of soil water depends on the threshold of soil water condition. Lamptey et al. (2020) and Snider et al. (2014) proved that the photosynthetic activity will be enhanced under moderate soil water condition but be lowered under excess water or severe water deficit conditions. In the present study, increased precipitation (W_+30_) did not exceed the threshold of soil moisture and significantly increased the value of *P*_n_. This suggests that the precipitation increased by 30% is a moderate soil water condition for the potential photosynthetic capacity of *P. australis*. The reduction of *P*_n_ at the W_−30_ condition demonstrated that the severe drought stress can drastically inhibit the photosynthesis of *P. australis*. At the same time, previous studies also showed that the photosynthesis and plant growth will be limited by higher temperature above the optimum point (Rodriguez et al., 2015). In our study, the reduction of *P*_n_ under T_4_ condition indicated that the temperature 4 °C higher than the ambient temperature (26.3/21.6 °C) has exceeded the optimum point and is adversely to the photosynthesis of *P. australis*. However, the threshold of soil water condition and the optimum temperature point for the photosynthesis of *P. australis* are still unclear and need further investigation. It is widely accepted that the decline in *P*_n_, *C*_i_, *T*_r_ and *WUE* could be attributed to decreased *G*_s_ under drought and heat stress conditions (Carvalho et al., 2019; Li et al., 2021a; Olorunwa, Shi & Barickman, 2021). In this study, under ambient temperature (T_0_) condition, the *G*_s_, *C*_i_, *T*_r_ and *WUE* showed no remarkable differences in different precipitation treatments, indicating the soil water deficit is not the limiting factor in stomatal openness, water consumption (transpiration) and utilization for *P. australis* plants. On the other hand, with the increasing of temperature (T_4_), precipitation decreased by 30% caused a remarkable reduction of *G*_s_, *C*_i_ and *T*_r_, suggesting that higher temperature exacerbates the detrimental effect of water shortage, which is in accordance with the studies on *Xanthoceras sorbifolium* Bunge (Du et al., 2021), *Solanum lycopersicum* (Raja et al., 2020), and *Stipa bungeana* (Song et al., 2016b). Furthermore, drought and heat stress also cause damage to the photosynthetic apparatus as confirmed by reduced *V*_cmax_ and *J*_max_, as the decline in these two parameters are ascribed to a reduced number of active Rubisco molecules and a decrease of photosynthetic energy during the process of CO_2_ assimilation (Olorunwa, Shi & Barickman, 2021; Zhuang et al., 2020).

**Figure 3:**
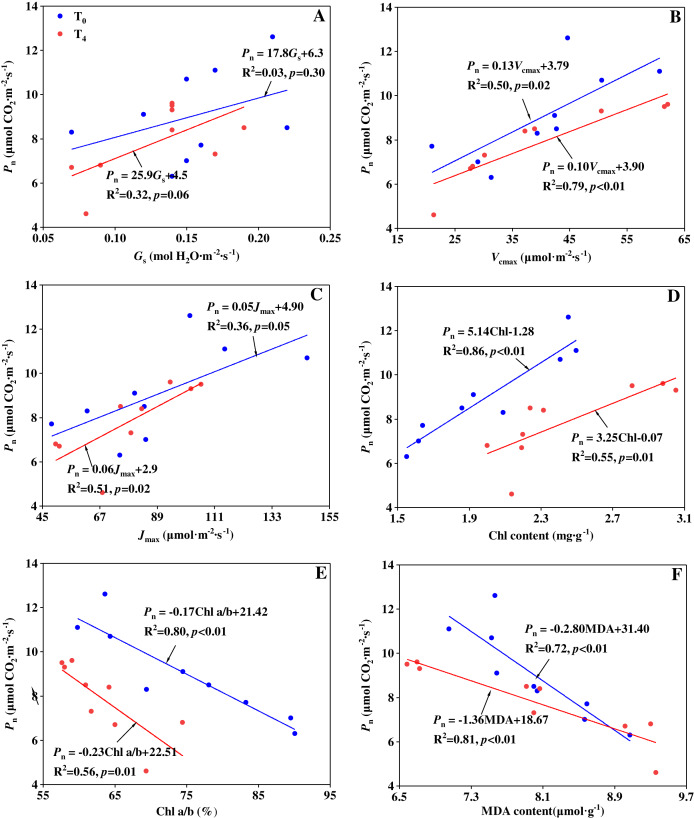
Relationship between *P*_n_ and (A) *G*_s_, (B) *V*_cmax_, (C) *J*_max_, (D) Chl a+b, (E) Chl a/b and (F) MDA content under ambient temperature (T_0_) and high temperature (T_4_) conditions.

The mechanisms of precipitation change affecting the carbon assimilation can be studied by stomatal limitation and non-stomatal limitation. Song et al. (2020) indicated that the reduction in photosynthesis of a water-stressed maize was mainly caused by stomatal limitation, whereas Li et al. (2020) reported that stomatal limitation did not play a major role in the change of photosynthesis of transgenic tobacco plants. The different results may be attributed to various responses from species, stress lasting time and stress intensity (Mitchell et al., 2008; Song et al., 2020). In our experiment, to figure out which is the main factor in limiting the photosynthesis, linear regression analysis was performed to illustrate the relationship of *P*_n_ with *G*_s_, *V*_cmax_, *J*_max_, Chl a+b content, Chl a/b ratio and MDA content under T_0_ and T_4_ conditions, respectively (Fig. 3). From the linear regression analyses, it was found there is no significant relationship between *P*_n_ and *G*_s_ (*p* > 0.05). But *P*_n_ had a significantly positive linear correlation with *V*_cmax_, *J*_max_ and Chl a+b content, as well as a significantly negative linear correlation with Chl a/b ratio and MDA content. The results indicate that non-stomatal limitation caused by precipitation change plays a major role in determining the carbon assimilation rate. Similar result can be found in the research by Xu & Zhou (2011), Song et al. (2016a), and Li et al. (2020). At the condition of T_0_, Chl a+b content had the closest relationship with *P*_n_ (R^2^ = 0.86, Fig. 3D) compared with other non-stomatal limitation factors. This suggests that the effect of increased precipitation on Chl content plays a major role in determining the carbon assimilation under ambient temperature condition. At the condition of T_4_, MDA content had the closest relationship with *P*_n_ (R^2^ = 0.81, Fig. 3F) compared with other non-stomatal limitation factors. This suggests that the effect of increased precipitation on lipid peroxidation plays a major role in determining the carbon assimilation under high temperature condition.

**Figure 4:**
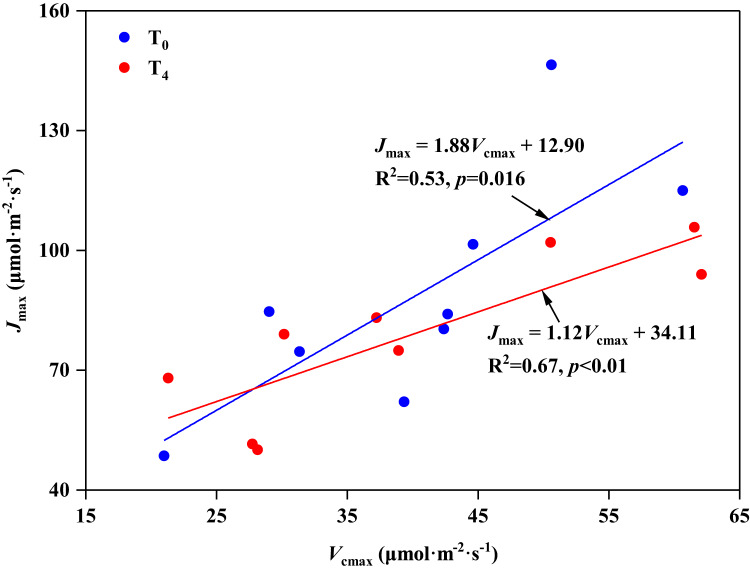
Relationship between the maximum rate of RuBP carboxylation (*V*_cmax_) and RuBP regeneration capacity (*J*_max_) in leaves of *Phragmites australis* under warming and precipitation change conditions.

In the present study, we found that high temperature induced the stomatal opening (increase in *G*_s_, Table 2), but resulted in a decrease in carbon assimilation (decrease in *P*_n_, Table 2), which is consistent with the research on *Leymus chinensis* by Xu & Zhou (2006). The response mechanism of plant photosynthesis to temperature can be studied by the balance between *V*_cmax_ and *J*_max_ (And & Sharkey, 1982; Song et al., 2016b). Wullschleger (1993) investigated 109 different species and concluded that there was a strong correlation between *V*_cmax_ and *J*_max_, which means there was a fixed balance relationship between RuBp carboxylation and regeneration in spite of the species or growth conditions. In our study, *V*_cmax_ and *J*_max_ showed a significant linear relationship under ambient temperature (T_0_) condition, with the ratio of *J*_max_ to *V*_cmax_ being 1.88 (*p* < 0.05, Fig. 4A). However, with the increasing of temperature (T_4_), even though there was still an obvious linear relationship between *V*_cmax_ and *J*_max_ (*p* < 0.05, Fig. 4B), the ratio of *J*_max_ to *V*_cmax_ decreased to 1.12. These results indicate that high temperature disrupted the balance between *V*_cmax_ and *J*_max_, resulting in a negative effect on the photosynthesis of *P. australis*. Similar results were also supported by the study of Huang et al. (2021).

**Figure 5:**
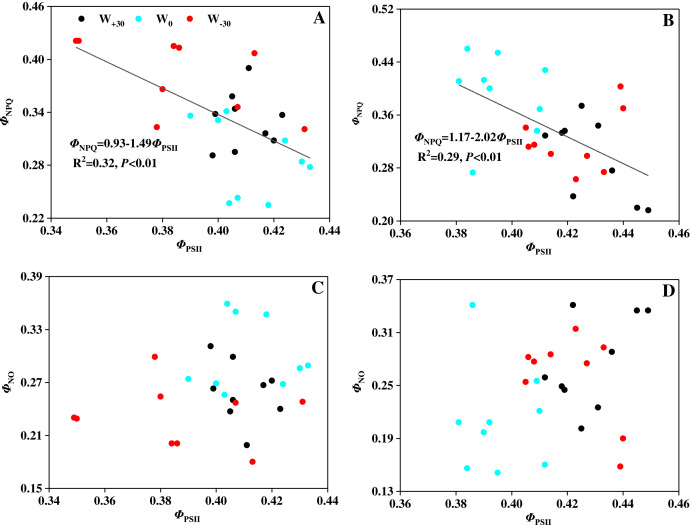
(A–D) Relationship between quantum yields of PSII photochemistry (*Φ*_PSII_) and quantum yields of regulated energy dissipation (*Φ*_NPQ_) in leaves of *Phragmites australis* under warming and precipitation change conditions.

Chlorophyll fluorescence can be used to detect the real photosynthetic behavior of the whole plant under stress quickly (Bhagooli et al., 2021). Based on this, it is possible to evaluate both the function of photosynthetic apparatus and the effects of environmental stress on plants. Environmental stress mainly damages the photosynthetic apparatus of PSII, and PSII will adjust the rate of electron transport and photochemical efficiency in response to the weakened ability of CO_2_ assimilation (Aragón-Gastélum et al., 2020; Hasanuzzaman et al., 2013). The damage caused by excess light energy to the system will be mitigated by heat dissipation. Water deficiency and heat stress will cause the inactivation or damage of leaf’s PSII reaction center (He et al., 2021; Mathur, Agrawal & Jajoo, 2014). This will lead to the damage of the photosynthetic apparatus and bring about the photoinhibition, which is consistent with the studies by Farfan-Vignolo & Asard (2012) and Yan et al. (2018). In our present research, precipitation change and high temperature had a significant effect on the photosynthesis of *P. australis*. However, how *P. australis* resists those environmental stresses to protect itself is still unknown. To solve this problem, three fluorescence parameters (*Φ*_PSII_, *Φ*_NPQ_ and *Φ*_NO_) based on Lake model were used to detect the partitioning of absorbed light energy and to explore the protective mechanism in PSII reaction center (Kramer et al., 2004; Li et al., 2019b). Among the three fluorescence parameters, *Φ*_PSII_ (absorbed light energy utilized by PSII photochemistry) reflects the linear electron transport indirectly, *Φ*_NPQ_ (thermally dissipated *via* ΔpH and xanthophyll-dependent energy quenching) represents the yield of dissipation by downregulation, and *Φ*_NO_ (thermally dissipated *via* ΔpH and xanthophyll-dependent energy quenching) reflects the yield of other non-photochemical losses (García-Sánchez et al., 2012; Nabi et al., 2021). In Figure 2, it was found that precipitation change and high temperature had no significant effect on the value of *Φ*_PSII_, suggesting that heat dissipation of the excess light energy was dissipated to the extracelular as a form of heat to protect the photosynthetic apparatus from damage caused by photoinhibition (Li et al., 2019a; Song et al., 2016b). Moreover, Figure 5 showed that there was a strong relationship (*p* < 0.01) between *Φ*_PSII_ and *Φ*_NPQ_, and the correlation between *Φ*_PSII_ and *Φ*_NO_ were not evident (*p* > 0.05). This suggests that the xanthophyll cycle-mediated thermal dissipation plays a major role in PSII photoprotection under changing precipitation and high temperature conditions, while the non-regulated quenching mechanism may play a less important role (Demmig-Adams & Adams, 2018; Stael et al., 2015). The results are opposite with the findings on plant responses to heat stress, water deficit and cold stress by other scholars (Dias et al., 2018; Osório et al., 2011; Savitch et al., 2009; Song, Wang & Lv, 2016). The possible reason is that *P. australis* as the dominant species of coastal wetlands in China, having a strong ability in resisting environmental stress by dissipating excess excitation energy, which cannot be used in PSII photochemistry reaction as harmless heat through the xanthophyll cycle (Demmig-Adams et al., 1996; Lu et al., 2020; Zhang et al., 2015).

## Conclusions

In conclusion, the photosynthesis of *P. australis* during precipitation changing is dependent on non-stomatal limitation but not stomatal closure, which have a significant negative linear correlation with Chl a/b ratio and MDA content. At the same time, high temperature causes the biochemical limitation on photosynthesis, inhibits the positive effects of increased precipitation and aggravates the adverse effects of drought on photosynthesis of *P. australis*. Even though high temperature and drought (precipitation decrease) significantly decrease the carbon assimilation rate, *P. australis* still has a strong ability to protect itself from damages by transforming excess excitation energy into harmless heat. This study highlighted the significant role of precipitation change in regulating the photosynthetic performance of *P*. *australis* under elevated temperature conditions, which may help us to better understand the mechanisms of vegetation degradation and provide knowledge basis for the restoration of the vegetation in climate sensitive regions under the background of global change.

## Supplemental Information

10.7717/peerj.13087/supp-1 Supplemental Information 1 Raw data. All measured parameters with three replicates.

## Additional Information and Declarations

### Data Availability

The following information was supplied regarding data availability: The raw measurements are available in the Supplemental File.
